# Predictors of Gross Hematuria After SARS-CoV-2 mRNA Vaccination in Patients with IgA Nephropathy

**DOI:** 10.34067/KID.0000000000000192

**Published:** 2023-06-09

**Authors:** Shinya Yokote, Nobuo Tsuboi, Akihiro Shimizu, Masahiro Okabe, Kotaro Haruhara, Takaya Sasaki, Hiroyuki Ueda, Takashi Yokoo

**Affiliations:** 1 Division of Nephrology and Hypertension, Department of Internal Medicine, Jikei University Katsushika Medical Center, Tokyo, Japan; 2 Division of Nephrology and Hypertension, Department of Internal Medicine, Jikei University School of Medicine, Tokyo, Japan; 3 Division of Nephrology and Hypertension, Department of Internal Medicine, Jikei University Kashiwa Hospital, Chiba, Japan; 4 Division of Nephrology and Hypertension, Department of Internal Medicine, Jikei University Daisan Hospital, Tokyo, Japan; 5 Division of Nephrology, Kawaguchi Municipal Medical Center, Kawaguchi, Japan

**Keywords:** COVID-19, drug interactions, glomerular disease, IgA nephropathy

## Abstract

**Key Points:**

- Little is known about the clinical characteristics of patients with immunoglobulin A nephropathy (IgAN) who present with gross hematuria in relation to SARS-CoV-2 mRNA vaccination.
- The relationship between the clinical features in patients with IgAN at the time of SARS-CoV-2 mRNA vaccination and the subsequent appearance of gross hematuria was investigated.
- This study demonstrates the clinical significance of microscopic hematuria in patients with IgAN as a predictor of gross hematuria after SARS-CoV-2 mRNA vaccination.

**Background:**

There have been several reports of immunoglobulin A nephropathy (IgAN) patients with gross hematuria and acute deterioration of urinary findings and kidney function after severe acute respiratory syndrome coronavirus 2 mRNA vaccination. Recent case series studies have indicated a possible link between the status of urinary findings at the time of vaccination and the subsequent appearance of gross hematuria. In this study, we aimed to determine whether the status of prevaccination urinary findings was associated with postvaccination gross hematuria in patients already diagnosed with IgAN.

**Methods:**

Outpatients with IgAN who had been followed up before vaccination were included. We analyzed the association between the remission of prevaccination microscopic hematuria (urine sediment <5 red blood cells/high-power field) or proteinuria (<0.3 g/gCr) and postvaccination gross hematuria.

**Results:**

A total of 417 Japanese patients with IgAN (median age, 51 years; 56% female; eGFR, 58 ml/min per 1.73 m^2^) were included. The frequency of gross hematuria after vaccination was higher in 20 of 123 patients (16.3%) with microscopic hematuria than in 5 of 294 patients (1.7%) without microscopic hematuria before vaccination (*P* < 0.001). There was no association between prevaccination proteinuria and postvaccination gross hematuria. After adjusting for potential confounders, such as sex (female), age (younger than 50 years), eGFR (≥60 ml/min per 1.73 m^2^), and histories of tonsillectomy and corticosteroid therapy, prevaccination microscopic hematuria was still associated with postvaccination gross hematuria (odds ratio, 8.98; *P* < 0.001). As the severity of prevaccination microscopic hematuria increased, the incidence of postvaccination gross hematuria increased (*P* < 0.001).

**Conclusions:**

Prevaccination microscopic hematuria in patients with IgAN is a major predictor of postvaccination gross hematuria, regardless of potential confounders, including previous treatments of IgAN.

## Graphical abstract

**Figure:**
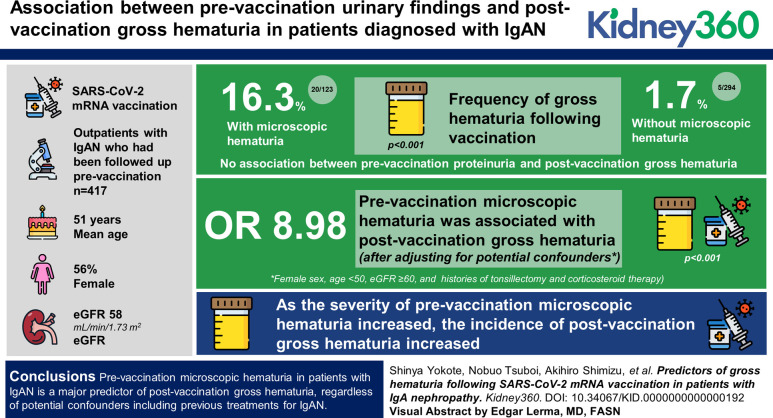

## Introduction

Since the commencement of mass-scale vaccination programs against the worldwide coronavirus disease 2019 pandemic, a series of newly diagnosed or exacerbated cases of immunoglobulin A nephropathy (IgAN) after severe acute respiratory syndrome coronavirus 2 (SARS-CoV-2) mRNA vaccination have been reported.^1–8^ Importantly, some IgAN patients with postvaccination gross hematuria may present as AKI, followed by CKD.^2,4–7^ Because repeated vaccinations are recommended for patients with glomerular diseases, including IgAN, identifying predictors of postvaccination gross hematuria is important. Notably, many of these reported IgAN cases showed persistent hematuria and/or proteinuria before SARS-CoV-2 mRNA vaccination,^3,4,8^ suggesting a possible link between the prevaccination status of urinary findings and postvaccination gross hematuria. However, to date, no previous study has examined the potential effect of prevaccination urinary findings on postvaccination gross hematuria in patients with IgAN.

This retrospective cohort study aimed to elucidate the clinical characteristics of patients with IgAN who were prone to gross hematuria after SARS-CoV-2 mRNA vaccination, primarily focusing on the prevaccination remission status of microscopic hematuria or proteinuria.

## Methods

### Study Design and Patients

Japanese patients with biopsy-proven IgAN who were older than 18 years and visited Jikei University Hospital, Jikei Katsushika Medical Center, Jikei Kashiwa Hospital, or Jikei Daisan Hospital from February 1, 2021, to September 20, 2021, were enrolled. The exclusion criteria were unvaccinated or unknown mRNA vaccination status, lack of urinalysis and blood tests before first vaccination, complications of other primary glomerular diseases, renal or urologic cancer, living kidney transplant recipients, eGFR <15 ml/min per 1.73 m^2^, and age younger than 18 years. This study was approved by the Ethics Review Board of the Jikei University School of Medicine (34-137 [11288]) and followed the tenets of the Declaration of Helsinki. Because this was a retrospective study, information about the research plan was posted; the patients were offered the opportunity to refuse participation; and individual informed consent was not required.

### Clinical Measurements

Clinical characteristics, including age, sex, body mass index, and medical history, including current use of renin-angiotensin-aldosterone system (RAAS) inhibitors and treatment history of corticosteroids or tonsillectomy, were obtained from the patient's medical records. Laboratory data from the last outpatient visit before vaccination, including serum creatinine (Cr), IgA, C3, urinary protein/Cr ratio (UPCR), and urinary sediments, were also obtained. eGFR was defined by the following formula for Japanese individuals: eGFR (ml/min per 1.73 m^2^)=194×Cr^−1.094^×age^−0.287^ (×0.739 for women).^9^ The red blood cell (RBC) count in the urinary sediment was graded as follows: 0, <5 RBCs/high-power field (HPF); 1, 5–19 RBCs/HPF; 2, 20–49 RBCs/HPF; and 3, ≥50 RBCs/HPF.^3^ Associations between the remission of microscopic hematuria (urine sediment <5 RBCs/HPF) or proteinuria (<0.3 g/gCr) before vaccination and gross hematuria after SARS-CoV-2 mRNA vaccination were analyzed. Laboratory data, including Cr, eGFR, UPCR, and urinary sediments, were collected not only before vaccination but also at the last outpatient visit after the appearance of gross hematuria. In analyses using a stricter definition of urinary remission, hematuria remission and proteinuria remission were defined by three consecutive negative urine tests performed at least 6 months before the first dose of the SARS-CoV-2 mRNA vaccine, on the basis of the criteria proposed by the Japanese Society of Nephrology.^10^ Information on the occurrence of gross hematuria was obtained through interviews and self-reports during the regular outpatient visits. eGFR slope (ΔeGFR) was defined as the change in eGFR between the outpatient visit immediately before vaccination and the most recent outpatient visit, divided by the number of days in the outpatient visit period and converted to 365 days. The percent change in eGFR (ΔeGFR%) was calculated from the percentage of ΔeGFR in eGFR at the outpatient visit immediately before vaccination.

### Statistical Analyses

Continuous variables are presented as median and interquartile range (IQR), and categorical variables are expressed as numbers (%). Nonparametric continuous variables for the two groups with correspondence were compared using the Wilcoxon signed-rank test, and those for the two groups without correspondence were compared using the Mann–Whitney *U* test. Categorical variables are presented as percentages and compared using Pearson chi-squared and Fisher exact tests. Clinically relevant factors identified by a subgroup comparison of patients and potential confounding factors, including treatment history for IgAN, such as corticosteroid treatment, RAAS inhibitor treatment, and tonsillectomy, were included in multivariate logistic regression analysis. The Cochran–Armitage test was used to determine whether there is a linear trend between the severity of prevaccine microscopic hematuria and the incidence of postvaccine gross hematuria. Statistical analyses were performed using the SPSS software package (version 29.0; IBM, Armonk, NY), GraphPad Prism (ver. 8.4.3; GraphPad Software, La Jolla, CA), and EZR software package (ver. 1.61; Saitama Medical Center, Jichi Medical University, Japan). Statistical significance was set at *P* < 0.05.

## Results

**Figure 1:**
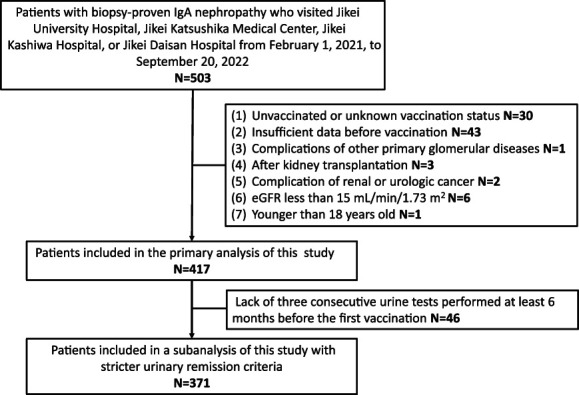
**Patient selection flowchart.** Overall, 503 patients with biopsy-proven immunoglobulin A nephropathy visited our facility from February 1, 2021, to September 20, 2022. Of these patients, 86 were excluded because of an unvaccinated or unknown vaccination status, lack of urine and blood tests before first vaccination, complications of other primary glomerular diseases, renal or urologic cancer, kidney transplantation, or age younger than 18 years. A total of 417 patients participated in the main analyses. Of these patients, 46 were excluded because of a lack of three consecutive urine tests performed at least 6 months before first vaccination. A total of 371 patients participated in the subanalyses using stricter criteria for urinary remission.

The patient selection process is shown in Figure 1. Of the 503 patients, 30 were unvaccinated or had an unknown vaccination status; 43 did not have sufficient data collection before the first dose of SARS-CoV-2 mRNA vaccination; one had other combined primary glomerular diseases; two had renal or urologic cancer; three were living kidney transplant recipients with IgAN; six had eGFR <15 ml/min per 1.73 m^2^; and one was younger than 18 years; hence, they were excluded. Finally, 417 patients with IgAN were included in this study.

Table 1 summarizes the clinical characteristics of the patients immediately before the first SARS-CoV-2 mRNA vaccination dose. Overall, the median age was 51 (IQR: 42–62) years, and 234 patients (56%) were female. The median history of IgAN was 12 (IQR: 5–21) years. There were no patients with suspected IgAN secondary to gastrointestinal or liver disease. Current treatment with RAAS inhibitors and a treatment history of corticosteroids or tonsillectomy were identified in 312 (75%), 225 (54%), and 169 (41%) patients, respectively. The median eGFR was 58 (IQR: 43–73) ml/min per 1.73 m^2^, and the median UPCR was 0.24 (IQR: 0.08–0.66) g/gCr. Before vaccination, microscopic hematuria and proteinuria were confirmed in 123 (29%) and 181 (43%) patients, respectively.

**Table 1:** Comparison of baseline clinical characteristics between patients with and without microscopic hematuria or proteinuria

Characteristics	Overall (*n*=417)	Patients without Microscopic Hematuria (*n*=294)	Patients with Microscopic Hematuria (*n*=123)	*P* Value	Patients without Proteinuria (*n*=236)	Patients with Proteinuria (*n*=181)	*P* Value
Age, yr	51 (42–62)	52 (43–63)	50 (37–60)	0.021	51 (42–62)	52 (40–63)	0.962
Female, *n* (%)	234 (56)	164 (56)	70 (57)	0.832	130 (55)	104 (57)	0.746
BMI, kg/m^2^	22.5 (20.1–24.8)	22.6 (20.3–24.8)	22.3 (19.9–24.9)	0.476	22.3 (20.0–24.3)	22.8 (20.3–25.3)	0.199
History of IgAN; yr	12 (5–21)	13 (6–22)	7 (2–16)	<0.001	11 (5–22)	11 (5–21)	0.763
Hypertension; *n* (%)	169 (41)	118 (40)	51 (41)	0.801	83 (35)	86 (48)	0.012
Diabetes; *n* (%)	29 (7)	21 (7)	8 (7)	0.815	14 (6)	15 (8)	0.355
eGFR; ml/min per 1.73 m^2^	58 (43–73)	56 (43–72)	63 (44–76)	0.055	61 (49–75)	50 (37–70)	<0.001
UPCR; g/gCr	0.24 (0.08–0.66)	0.18 (0.07–0.48)	0.38 (0.18–0.98)	<0.001	0.10 (0.05–0.17)	0.77 (0.45–1.26)	<0.001
Hematuria grade				<0.001			<0.001
0–4 RBCs/HPF; *n* (%)	294 (71)	294 (100)	0 (0)		186 (79)	108 (60)	
5–19 RBCs/HPF; *n* (%)	72 (17)	0 (0)	72 (58)		30 (13)	42 (23)	
20–49 RBCs/HPF; *n* (%)	27 (6)	0 (0)	27 (22)		12 (5)	15 (8)	
50–Many RBCs/HPF; *n* (%)	24 (6)	0 (0)	24 (20)		8 (3)	16 (9)	
Serum IgA^a^; mg/dl	269 (201–362)	261 (198–348)	305 (223–398)	0.006	268 (198–353)	270 (212–371)	0.251
Serum C3^a^; mg/dl	99 (87–113)	97 (87–113)	102 (88–113)	0.504	97 (85–112)	102 (89–115)	0.025
RAAS inhibitor use; *n* (%)	312 (75)	222 (76)	90 (73)	0.616	157 (67)	155 (86)	<0.001
Corticosteroid use; *n* (%)	225 (54)	162 (55)	63 (51)	0.519	122 (52)	103 (57)	0.334
Tonsillectomy; *n* (%)	169 (41)	120 (41)	49 (40)	0.902	94 (40)	75 (41)	0.795

Baseline clinical characteristics were evaluated at the last visit before the first vaccination dose. Values for categorical variables are presented as numbers (percentages); values for continuous variables are given as medians (interquartile ranges). Nonparametric continuous variables were compared using the Mann–Whitney *U* test. Categorical variables are presented as percentages and compared using the Pearson chi-square test and Fisher exact test. BMI, body mass index; IgAN, immunoglobulin A nephropathy; UPCR, urine protein/creatinine ratio; Cr, serum creatinine; RBC, red blood cell; HPF, high-power field; RAAS, renin-angiotensin-aldosterone system.

a Because serum IgA and C3 values were frequently missing, the results of analyses of 310 and 300 patients, respectively, are shown.

**Figure 2:**
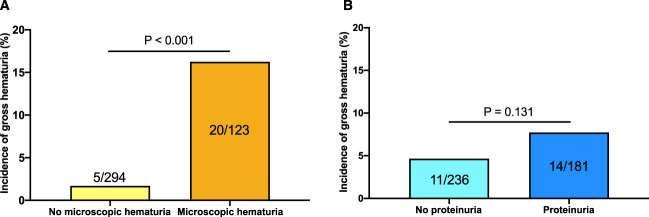
**The incidence of postvaccination gross hematuria.** (A) The incidence of postvaccination gross hematuria in patients with or without prevaccination microscopic hematuria and (B) in patients with or without prevaccination proteinuria.

Table 1 presents the subgroup analysis comparing background factors between the patient groups categorized on the basis of the presence or absence of prevaccination microscopic hematuria or proteinuria. The results showed several variables that may confound prevaccination microscopic hematuria or proteinuria regarding postvaccination gross hematuria. In this study population, the incidence of postvaccination gross hematuria was 5 of 294 (1.7%) in patients without prevaccination microscopic hematuria and 20 of 123 (16.3%) in patients with prevaccination microscopic hematuria, showing a significant difference among the groups (Figure 2A, *P* < 0.001). By contrast, there was no significant difference in the incidence of postvaccination gross hematuria between patients with and without prevaccination proteinuria (Figure 2B).

**Figure 3:**
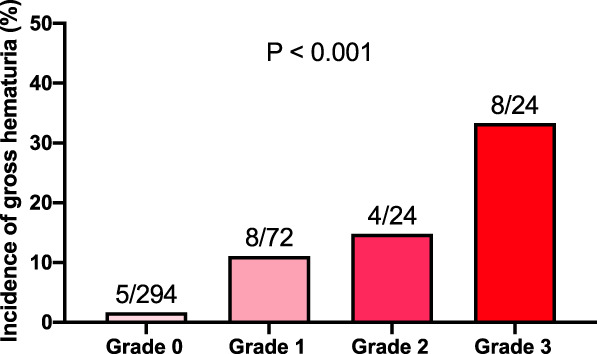
**Correlation between severity of prevaccination microscopic hematuria and incidence of postvaccination gross hematuria.** The Cochran–Armitage test showed a linear trend between the severity of prevaccination microscopic hematuria and the incidence of postvaccination gross hematuria (*P* < 0.001).

Table 2 shows the results of univariable and multivariable logistic analyses of factors related to postvaccination gross hematuria. In univariable analysis, age younger than 50 years, female sex, IgAN for over 10 years after diagnosis, eGFR ≥60 ml/min per 1.73 m^2^, and prevaccination microscopic hematuria were identified as factors associated with postvaccination gross hematuria. In multivariable analysis, female sex and prevaccination microscopic hematuria in model 1 were identified as significant factors associated with postvaccination gross hematuria (odds ratio [OR], 3.17; 95% confidence interval [CI]: 1.12 to 8.97; *P* < 0.030, OR, 10.30; 95% CI, 3.64 to 29.11; *P* < 0.001, respectively). After adjusting for potential confounders, such as sex (female), age ( younger than 50 years), eGFR (≥60 ml/min per 1.73 m^2^), and histories of tonsillectomy and corticosteroid therapy, prevaccination microscopic hematuria was still associated with postvaccination gross hematuria (OR, 8.98; 95% CI, 3.08 to 26.17; *P* < 0.001). Furthermore, higher the degree of microscopic hematuria before vaccination, greater the risk of postvaccination gross hematuria (Figure 3, *P* < 0.001).

**Table 2:** Univariable and multivariable analyses of variables associated with gross hematuria after severe acute respiratory syndrome coronavirus 2 mRNA vaccination

Variables	Univariable			Multivariable								
				Model 1			Model 2			Model 3		
	OR	95% CI	*P* Value	OR	95% CI	*P* Value	OR	95% CI	*P* Value	OR	95% CI	*P* Value
Microscopic hematuria	11.22	4.11 to 30.68	<0.001	10.03	3.64 to 29.11	<0.001	8.74	3.04 to 25.13	<0.001	8.98	3.08 to 26.17	<0.001
Proteinuria ≥0.3 g/gCr	1.89	0.82 to 4.35	0.131	1.16	0.47 to 2.86	0.743	1.21	0.48 to 3.02	0.688	1.12	0.44 to 2.86	0.818
Age <50 yr	1.88	0.82 to 4.29	0.128	1.67	0.68 to 4.11	0.262	0.96	0.33 to 2.75	0.934	1.22	0.38 to 3.89	0.739
Female	3.33	1.22 to 9.04	0.013	3.17	1.12 to 8.97	0.030	2.87	1.00 to 8.22	0.049	2.93	0.99 to 8.61	0.050
BMI ≧25 kg/m^2^	0.63	0.21 to 1.87	0.398									
History of IgAN ≧10 yr	0.35	0.15 to 0.84	0.014				0.63	0.24 to 1.66	0.348	0.59	0.22 to 1.57	0.287
Hypertension	0.55	0.23 to 1.35	0.188									
Diabetes	—	—	0.242									
eGFR ≧60 ml/min per 1.73 m^2^	3.87	1.51 to 9.90	0.003				2.49	0.81 to 7.61	0.110	2.61	0.86 to 7.92	0.089
IgA ≧300 mg/dl	0.54	0.20 to 1.42	0.205									
RAAS inhibitor use	0.58	0.25 to 1.35	0.199							1.23	0.43 to 3.54	0.699
Corticosteroid use	0.77	0.34 to 1.73	0.529							1.23	0.42 to 3.59	0.705
Tonsillectomy	0.67	0.28 to 1.60	0.365							0.47	0.15 to 1.47	0.192

Model 1: Age, sex, prevaccination microscopic hematuria, and prevaccination proteinuria.

Model 2: Age, sex, prevaccination microscopic hematuria, prevaccination proteinuria, eGFR, and history of IgAN.

Model 3: Age, sex, prevaccination microscopic hematuria, prevaccination proteinuria, eGFR, history of IgAN, RAAS inhibitor use, corticosteroid use, and tonsillectomy.

Categorical variables are presented as percentages and compared using the Pearson chi-square test and Fisher exact test. Clinically relevant factors identified by a subgroup comparison of patients and potential confounding factors, including treatment history for IgAN, such as corticosteroid treatment, renin-angiotensin-aldosterone system inhibitor treatment, and tonsillectomy, were included in the multivariate logistic regression analyses. OR, odds ratio; CI, confidence interval; Cr, serum creatinine; BMI, body mass index; IgAN, immunoglobulin A nephropathy; RAAS, renin-angiotensin-aldosterone system.

A sensitivity analysis with a subgroup of male patients, which was performed to exclude the possibility of false-positive results in identifying microscopic hematuria because of menstruation in female patients, yielded results almost identical to the main results (Supplemental Table 1). Prevaccination microscopic hematuria was still identified as a significant factor associated with postvaccination gross hematuria in a subgroup of male patients (OR, 13.19; 95% CI, 1.23 to 141.92; *P* = 0.033). Hematuria and proteinuria often fluctuate, and a single-point evaluation may not be sufficient to determine remission. A sensitivity analysis was, therefore, performed using a stricter criteria for the remission of hematuria and proteinuria defined by three consecutive negative urine tests performed at least 6 months before the first dose of the SARS-CoV-2 mRNA vaccine,^10^ yielding results almost identical to the main results (OR, 6.70; 95% CI, 2.12 to 21.16; *P* = 0.001) (Supplemental Table 2).

Supplemental Table 3 summarizes the clinical characteristics of the 25 patients with postvaccination gross hematuria. As in previously reported cases, patients were predominantly female (80%),^3,8^ and most patients (72%) presented with gross hematuria after the second or subsequent vaccination dose.^2–4,8^ Of the 25 patients with postvaccination gross hematuria, 12 patients had received the Pfizer vaccine, ten patients had received the Moderna vaccine, one patient had received both vaccines, and two patients had received mRNA vaccines from unknown companies. None of the patients with postvaccination gross hematuria in this study received a bivalent vaccine. There were no significant differences in RAAS inhibitor use, corticosteroid use, or tonsillectomy before vaccination between the patients with and without gross hematuria. None of the patients with IgAN who presented with postvaccination gross hematuria underwent a repeat kidney biopsy. Owing to gross hematuria after vaccination, two patients underwent tonsillectomy, and four patients received corticosteroid pulse therapy. As for the overall trend, the degree of ΔeGFR% in patients with postvaccination gross hematuria was significantly greater than that in patients without postvaccination gross hematuria (−4.08 versus −1.91%/yr; *P* = 0.021) (Supplemental Table 4). No patients with postvaccination hematuria developed AKI (elevated Cr >0.3), and eight patients (32%) had significant proteinuria (UPCR >1.0 g/gCr). Of the patients with postvaccination gross hematuria, no obvious differences were identified in prevaccination clinical findings between the two groups classified according to the degree of ΔeGFR% (Supplemental Table 5). Univariable and multivariable logistic analyses of factors related to greater percent change in eGFR (ΔeGFR% < −2.0%/yr) were performed for the cohort as a whole (Supplemental Table 6). Postvaccination gross hematuria was identified as a significant variable in the univariable analysis but not in the multivariable analysis.

## Discussion

This study examined the association between the prevaccination urinalysis status and the incidence of postvaccination gross hematuria in patients with IgAN. Our results clearly showed that prevaccination microscopic hematuria in patients with IgAN was associated with postvaccination gross hematuria, even after adjusting for potential confounding factors, including age, sex, duration of IgAN, kidney function, and treatment history for IgAN, such as RAAS inhibitor use, corticosteroid use, and tonsillectomy. Prevaccination proteinuria was not associated with postvaccination gross hematuria in the same cohort.

A subset of patients with IgAN presents with gross hematuria after an acute infection by microorganisms through the upper respiratory tract or intestines. A history of gross hematuria has been reported to predict a better prognosis for IgAN.^11^ However, the significantly younger age of the patients with gross hematuria suggests that these patients may simply have had shorter disease duration, which may represent a lead-time bias.^12^ Importantly, previous studies have shown that patients with IgAN with postinfectious gross hematuria may develop AKI, which leads to CKD in some patients.^13–15^ Similarly, an increasing number of patients with worsening kidney function because of gross hematuria after SARS-CoV-2 mRNA vaccination have been reported, with some patients developing CKD, requiring aggressive immunosuppressive therapy and/or dialysis therapy.^2,4,16^ Although the short-term clinical course in patients with IgAN who received SARS-CoV-2 mRNA vaccines has been reported to be generally favorable,^17^ the disease outcomes of IgAN patients with gross hematuria after SARS-CoV-2 mRNA vaccination are unclear. It is noteworthy that patients with IgAN with postvaccination gross hematuria in our cohort showed a slight worsening of kidney function compared with baseline values before vaccination. In addition, a clinical study recently reported that a second or third vaccination is associated with a two-fold increased risk of a flare-up in patients with IgAN,^18^ and there is concern that the repeated stimulation of the immune system by SARS-CoV-2 mRNA vaccination may further increase the frequency of recurrence in patients with glomerular diseases.^19^ Therefore, the indication for repeated SARS-CoV-2 mRNA vaccinations in patients with IgAN should be carefully determined individually, considering multiple factors, including the severity of microscopic hematuria and the history of postvaccination gross hematuria.

At present, the molecular mechanisms responsible for postvaccination gross hematuria in patients with IgAN remain unclear, but recent studies indicate the possible involvement of several immunological disorders in the pathogenesis. SARS-CoV-2 mRNA vaccination has been reported to stimulate toll-like receptors^20^ and may lead to increased production of Gd-IgA1, which is known to be preferentially deposited in glomeruli in patients with IgAN.^21^ Furthermore, SARS-CoV-2 mRNA vaccines have been shown to induce complement activation–related pseudo allergy (CARPA).^22,23^ The mRNA vaccines contain polyethylene glycol (PEG) as a PEG-micellar carrier system. CARPA is considered a non–IgE-mediated reaction and partly attributed to the binding of preexisting anti-PEG IgM to the liposomes with subsequent complement activation, which may have contributed to the exacerbation of IgAN.^24^ Female patients are more likely to be exposed to PEG through cosmetics. The greater prevalence of postvaccination gross hematuria in female patients may be in part due to CARPA-mediated exacerbation of IgAN.

Patients with prevaccination microscopic hematuria had shorter disease duration and higher proteinuria levels, potentially indicating ongoing chronic active glomerulonephritis from their IgAN. Considering the relationship between prevaccination microscopic hematuria and postvaccination gross hematuria shown in this study, it is possible that IgAN patients with microscopic hematuria are more susceptible to such immunological stimuli and exacerbation of IgAN. Consistent with this idea, this study showed that higher the degree of microscopic hematuria before vaccination, greater the risk of postvaccination gross hematuria. On the other hand, the pathogenesis of proteinuria may be complicated by the influence of chronic sclerosing lesions and the activity of glomerulonephritis. Therefore, comparisons on the basis of the presence or absence of proteinuria before vaccination may not have revealed a significant difference in the incidence of postvaccination gross hematuria. Future studies are, therefore, needed to compare changes in the above-described immunologic indices after SARS-CoV-2 mRNA vaccination in IgAN patients with or without prevaccination hematuria.

This study was the first to focus on the clinical characteristics of patients with IgAN, particularly urinary findings before SARS-CoV-2 mRNA vaccination and postvaccination gross hematuria. However, this study has notable limitations. First, information on the presence or absence of postvaccination gross hematuria relied on self-reporting, which does not ensure accuracy. Second, ΔeGFR% was greater in patients with postvaccination gross hematuria than in those without postvaccination gross hematuria. However, for the cohort as a whole, no significant relationship was identified between postvaccination gross hematuria and the percent change in eGFR, after adjustment for clinical confounders that may be related to renal prognosis. Therefore, we cannot conclude from this study that postvaccination gross hematuria in patients with IgAN affects renal prognosis. Finally, gross hematuria in our cohort was restricted to that related to SARS-CoV-2 mRNA vaccination. Therefore, microscopic hematuria may not be widely generalizable as a risk factor of postinfectious gross hematuria in patients with IgAN.

In conclusion, this study provides novel evidence that microscopic hematuria before SARS-CoV-2 mRNA vaccination is a major predictor of postvaccination gross hematuria in patients with IgAN. Gross hematuria in patients with IgAN may have a risk of inducing acute or, in some cases, irreversible, CKD. Thus, such information regarding the characteristic clinical findings of patients with IgAN prone to gross hematuria would be helpful when considering repeated SARS-CoV-2 mRNA vaccinations among patients with IgAN with clinically divergent phenotypes. Further studies on gross hematuria in patients with IgAN may provide important clues for understanding the pathogenesis of IgAN.

## Disclosures

K. Haruhara reports the following: Research Funding: Takeda Science Foundation. M. Okabe reports the following: Honoraria: AstraZeneca K.K., Chugai Pharmaceutical Co., Ltd., Kissei Pharmaceutical Co., Ltd, Kyowa Kirin Co., Ltd., Mitsubishi Tanabe Pharma Corporation. T. Yokoo reports the following: Advisory or Leadership Role: Editorial Board Member of “HUMAN CELL”; Board of Directors of Japanese Society of Nephrology. All remaining authors have nothing to disclose.

## Funding

None.

## Author Contributions

**Conceptualization:** Shinya Yokote.

**Data curation:** Kotaro Haruhara, Masahiro Okabe, Akihiro Shimizu, Nobuo Tsuboi, Hiroyuki Ueda, Shinya Yokote.

**Formal analysis:** Kotaro Haruhara, Masahiro Okabe, Takaya Sasaki, Akihiro Shimizu, Nobuo Tsuboi, Hiroyuki Ueda, Shinya Yokote.

**Investigation:** Kotaro Haruhara, Masahiro Okabe, Akihiro Shimizu, Hiroyuki Ueda, Shinya Yokote.

**Methodology:** Kotaro Haruhara, Masahiro Okabe, Akihiro Shimizu, Hiroyuki Ueda, Shinya Yokote.

**Project administration:** Shinya Yokote.

**Supervision:** Nobuo Tsuboi, Takashi Yokoo.

**Visualization:** Nobuo Tsuboi.

**Writing – original draft:** Nobuo Tsuboi, Shinya Yokote.

**Writing – review & editing:** Kotaro Haruhara, Masahiro Okabe, Takaya Sasaki, Akihiro Shimizu, Nobuo Tsuboi, Hiroyuki Ueda.

## Supplementary Material

## Supplemental Material

Supplemental Table 1. Univariable and multivariable analyses of variables associated with gross hematuria in a subgroup of male patients.

Supplemental Table 2. Univariable and multivariable analyses of variables associated with gross hematuria after SARS-CoV-2 mRNA vaccination using a stricter definition of urinary remission.

Supplemental Table 3. Clinical characteristics of 25 patients with gross hematuria after SARS-CoV-2 mRNA vaccination.

Supplemental Table 4. Comparison of ΔeGFR% between patients with and without postvaccination gross hematuria.

Supplemental Table 5. Comparison of baseline clinical characteristics between patients with postvaccination gross hematuria classified according to the ΔeGFR%

Supplemental Table 6. Univariable and multivariable logistic analyses of factors related to greater percent change in eGFR (ΔeGFR% < −2.0%/yr).
