# Supplementary material for: Predictors of Gross Hematuria After SARS-CoV-2 mRNA Vaccination in Patients with IgA Nephropathy
Source: Kidney360. 2023 Jun 9;4(7):943–50. doi: 10.34067/KID.0000000000000192 (PMC10371300; doi:10.34067/KID.0000000000000192)
Supplement: Supplementary file 1 [file kidney360-4-0943-s001.pdf]

Supplementary materials

Supplementary Table S1. Univariable and multivariable analyses of variables associated with gross hematuria in a subgroup of male patients

Variables	Univariable			Multivariable					
				Model 1			Model 2		
	OR	95% CI	*P* value	OR	95% CI	*P* value	OR	95% CI	*P* value
Microscopic hematuria	10.53	1.15–96.56	0.025	12.80	1.31–125.54	0.029	13.19	1.23–141.92	0.033
Proteinuria $\geq$ 0.3g/gCr	0.90	0.15–5.49	1.000	0.49	0.07-3.29	0.460	0.38	0.05–2.83	0.346
Age < 50 years	2.31	0.38–14.20	0.389	2.19	0.34–13.98	0.409	2.56	0.32–20.30	0.372
BMI ≧ 25 kg/m^2	0.73	0.08–6.73	1.000						
History of IgA nephropathy ≧ 10 years	0.41	0.07–2.53	0.378						
Hypertension	1.72	0.28–10.53	0.667						
Diabetes	-	-	1.000						
eGFR ≧ 60 mL/min/1.73 m^2	2.37	0.39–14.54	0.383						
IgA ≧ 300 mg/dL	-	-	0.125						
RAAS inhibitor use	-	-	1.000				-	-	0.998
Corticosteroid use	-	-	0.064				-	-	0.996
Tonsillectomy	2.31	0.38–14.20	0.389				0.78	0.10–6.10	0.813

Model 1: Age, pre-vaccination microscopic hematuria, pre-vaccination proteinuria.

Model 2: Age, pre-vaccination microscopic hematuria, pre-vaccination proteinuria,

RAAS inhibitor use, corticosteroid use, and tonsillectomy.

In each model, multivariate analysis was performed using clinically important factors. Abbreviations: BMI, body mass index; CI, confidence interval; eGFR, estimated glomerular filtration rate; HPF, high-power field; OR, odds ratio; RAAS, renin-angiotensin-aldosterone system; UPCR, urinary protein-to-creatinine ratio.

Supplementary Table S2. Univariable and multivariable analyses of variables associated with gross hematuria following SARS-CoV-2 mRNA vaccination using a stricter definition of urinary remission

Variables	Univariable			Multivariable								
				Model 1			Model 2			Model 3		
	OR	95% CI	*P* value	OR	95% CI	*P* value	OR	95% CI	*P* value	OR	95% CI	*P* value
No remission of microscopic hematuria	10.10	3.39–30.10	< 0.001	8.55	2.82–25.96	< 0.001	7.25	2.35–22.42	< 0.001	6.70	2.12–21.16	0.001
No remission of proteinuria	1.93	0.81–4.58	0.133	1.29	0.51–3.24	0.594	1,32	0.51–3.43	0.568	1.37	0.52–3.60	0.529
Age < 50 years	2.08	0.91–4.76	0.078	1.67	0.70–4.00	0.250	0.83	0.30–2.33	0.723	0.99	0.31–3.12	0.985
Female	3.78	1.39–10.29	0.006	3.18	1.13–8.95	0.029	2.81	0.98–8.03	0.054	2.69	0.93–7.74	0.068
BMI ≧ 25 kg/m^2	0.56	0.19–1.67	0.288									
History of IgAN ≧ 10 years	0.36	0.15–0.85	0.016				0.65	0.24–1.72	0.384	0.67	0.25–1.80	0.427
Hypertension	0.50	0.20–1.22	0.120									
Diabetes	-	-	0.242									
eGFR ≧ 60 mL/min/1.73 m^2	4.55	1.77–11.68	< 0.001				3.26	1.07–9.92	0.038	3.24	1.07–9.83	0.038

RAAS inhibitor use	0.42	0.18–0.99	0.042				0.85	0.31–2.33	0.757
Corticosteroid use	0.73	0.33–1.65	0.450				0.99	0.34–2.87	0.981
Tonsillectomy	0.68	0.29–1.63	0.389				0.57	0.19–1.78	0.336

Model 1: Age, sex, no remission of microscopic hematuria, no remission of proteinuria.

Model 2: Age, sex, no remission of microscopic hematuria, no remission of proteinuria, eGFR, and history of IgAN.

Model 3: Age, sex, no remission of microscopic hematuria, no remission of proteinuria, eGFR, history of IgAN, RAAS inhibitor use, corticosteroid use, and tonsillectomy.

Microscopic hematuria remission and proteinuria remission were defined based on three consecutive negative urine tests during at least 6 months prior to the 1st dose vaccination as described previously.[10] Categorical variables are presented as percentages and compared using Pearson's chi-square test and Fisher's exact test. Clinically relevant factors identified by subgroup comparison of patients and potential confounding factors, including treatment history for IgAN, such as corticosteroid treatment, renin-angiotensin-aldosterone system

inhibitor treatment, and tonsillectomy, were included in the multivariate logistic regression analyses.

Abbreviations: BMI, body mass index; CI, confidence interval; eGFR, estimated glomerular filtration rate; HPF, high-power field; IgAN, IgA nephropathy; OR, odds ratio; RAAS, renin-angiotensin-aldosterone system.

Supplementary Table S3. Clinical characteristics of 25 patients with gross hematuria following SARS-CoV-2 mRNA vaccination

Clinical characteristics	Patients with gross hematuria (n=25)	Patients without gross hematuria (n=392)	*P* value
Age; years	46［32–54］	52［42–63］	0.012
Female; n (%)	20 (80)	214 (55)	0.013
BMI; kg/m^2	21.1［18.2–23.9］	22.5［20.1–24.8］	0.048
History of IgA nephropathy; years	5［2–12］	12［5–21］	0.003
Hypertension; n (%)	7 (28)	162 (41)	0.188
Diabetes; n (%)	0 (0)	29 (7)	0.242
RAAS inhibitor use; n (%)	16 (64)	296 (76)	0.199
Corticosteroid use; n (%)	12 (48)	213 (54)	0.529
Tonsillectomy; n (%)	8 (32)	161 (41)	0.365
mRNA vaccines associated with gross hematuria			
Pfizer BNT162b2	12 (48)		
Moderna mRNA-1273	10 (40)		
Pfizer and Moderna	1 (4)		
Unknown	2 (8)		
Number of vaccinations at the time of initial gross hematuria	2［2–3］		
First; n (%)	7 (28)		

Second; n (%)	10 (40)		
Third; n (%)	7 (28)		
Fourth; n (%)	1 (4)		
Days from vaccination to the appearance of gross hematuria	1 [1–2]		
Other adverse events			
Fever; °C	38.0 [37.7–38.6]		
Headache; n (%)	10 (40)		
Fatigue; n (%)	14 (56)		
Arm pain; n (%)	12 (48)		
eGFR; mL/min/1.73 m^2	71 [60–84]	57 [43–73]	0.002
Serum IgA*; mg/dL	259 [201–340]	271 [201–363]	0.508
Serum C3*; mg/dL	91 [84–106]	99 [87–113]	0.173
UPCR; g/gCr	0.46 [0.13–0.88]	0.24 [0.08–0.61]	0.235
Hematuria grade			< 0.001
0–4 RBC/HPF; n (%)	5 (20)	289 (74)	
5–19 RBC/HPF; n (%)	8 (32)	64 (16)	
20–49 RBC/HPF; n (%)	4 (16)	23 (6)	
50–many RBC/HPF; n (%)	8 (32)	16 (4)	

Values for categorical variables are presented as numbers (percentages); values for continuous variables are given as medians [interquartile ranges]. * Serum IgA and C3 values were frequently missing, and the results from the analyses of 310 and 300 cases

are shown, respectively. Abbreviations: BMI, body mass index; eGFR, estimated glomerular filtration rate; HPF, high power field; RAAS, renin-angiotensin-aldosterone system; RBC, red blood cells; UPCR, urine protein/creatinine ratio.

Supplementary Table S4. Comparison of ΔeGFR% between patients with and without post-vaccination gross hematuria

	**Patients with gross hematuria (n=24)**	**Patients without gross hematuria (n=392)**	***P* value**
Observation period	607 ［534–647］	595 ［546–644］	0.745
eGFR just before vaccination; mL/min/1.73m^2	70 ［60–83］	57 ［43–73］	0.004
Most recent eGFR; mL/min/1.73m^2	63 ［56–70］	55 ［41–71］	0.063
ΔeGFR; mL/min/1.73m^2 per year	-2.55 ［-7.95 – -0.22］	-1.03 ［-3.00 – 1.16］	0.010
ΔeGFR%; % per year	-4.08 ［-10.28 – -0.37］	-1.91 ［-5.88 – 2.42］	0.021

Of the 25 patients with gross hematuria, 24 were analyzed, excluding one patient who subsequently became pregnant. eGFR slope (ΔeGFR) was defined as the change in eGFR between the outpatient visit immediately before vaccination and the most recent outpatient visit, divided by the number of days in the outpatient visit period and converted to 365 days. The percent change in eGFR (ΔeGFR%) was calculated from the percentage of ΔeGFR in eGFR at the outpatient visit immediately before vaccination. Values for continuous variables are given as medians [interquartile ranges].

Abbreviations: eGFR, estimated glomerular filtration rate.

Supplementary Table S5. Comparison of baseline clinical characteristics between patients with post-vaccination gross hematuria classified according to the ΔeGFR%

Characteristics	Low ΔeGFR% group (n=12)	High ΔeGFR% group (n=12)	*P* value
ΔeGFR%; % per year	-10.29 [-12.66 – -7.94]	0.05 [-3.11–2.82]	< 0.001
Age; years	41 [32–53]	50 [43–54]	0.443
Female; n (%)	10 (83)	9 (75)	1.000
BMI; kg/m^2	22.8 [19.8–24.2]	20.0 [18.0–21.4]	0.266
History of IgA nephropathy; years	4 [1–8]	7 [4–13]	0.178
Hypertension; n (%)	3 (25)	4 (33)	1.000
Diabetes; n (%)	0 (0)	0 (0)	-
eGFR; mL/min/1.73 m^2	77 [67–85]	64 [58–74]	0.266
UPCR; g/gCr	0.64 [0.13–1.27]	0.32 [0.12–0.56]	0.288
Hematuria grade			0.060
0–4 RBC/HPF; n (%)	2 (17)	3 (25)	
5–19 RBC/HPF; n (%)	1 (8)	7 (58)	
20–49 RBC/HPF; n (%)	4 (33)	0 (0)	
50–many RBC/HPF; n (%)	5 (42)	2 (17)	
Serum IgA*; mg/dL	240 [214–259]	261 [196–373]	0.710
Serum C3*; mg/dL	101 [91–107]	87 [80–91]	0.113
RAAS inhibitor use; n (%)	7 (58)	9 (75)	0.667

Corticosteroid use; n (%)	5 (42)	6 (50)	0.682
Tonsillectomy; n (%)	3 (25)	4 (33)	1.000

Of the 25 patients with gross hematuria, 24 were analyzed, excluding one patient who subsequently became pregnant. The percent change in eGFR (ΔeGFR%) was calculated from the percentage of ΔeGFR in eGFR at the outpatient visit immediately before vaccination. Twenty-four patients were analyzed by dividing them into two groups based on the median ΔeGFR%. Values for categorical variables are presented as numbers (percentages); values for continuous variables are given as medians (interquartile ranges). Nonparametric continuous variables were compared using the Mann–Whitney U test. Categorical variables are presented as percentages and compared using Pearson's chi-square test and Fisher's exact test. Abbreviations: BMI, body mass index; Cr, serum creatinine; eGFR, estimated glomerular filtration rate; HPF, high power field; RAAS, renin-angiotensin-aldosterone system; RBC, red blood cells; UPCR, urine protein/creatinine ratio.

Supplementary Table S6. Univariable and multivariable logistic analyses of factors related to greater percent change in eGFR (ΔeGFR% < -2.0 % per year)

Variables	Univariable			Multivariable		
	OR	95% CI	*P*-value	OR	95% CI	*P*-value
Microscopic hematuria	1.00	0.66–1.53	1.000	0.69	0.43–1.11	0.126
Proteinuria ≥ 0.3g/gCr	1.98	1.33–2.94	< 0.001	2.11	1.36–3.25	< 0.001
Post-vaccination gross hematuria	2.56	1.04–6.30	0.035	2.44	0.92–6.46	0.072
Age < 50 years	1.00	0.68–1.47	1.000	0.81	0.48–1.35	0.413
Female	1.19	0.81–1.76	0.374	1.11	0.74–1.69	0.610
BMI ≧ 25 kg/m^2	0.95	0.60–1.50	0.814	0.89	0.53–1.48	0.641
History of IgAN ≧ 10 years	0.66	0.45–0.98	0.038	0.68	0.44–1.05	0.081
Hypertension	1.25	0.84–1.84	0.272	1.18	0.73–1.91	0.494
Diabetes	0.80	0.38–1.71	0.564	0.75	0.33–1.70	0.487
eGFR ≧ 60 mL/min/1.73 m^2	1.03	0.70–1.52	0.880	1.19	0.74–1.91	0.472
RAAS inhibitor use	1.23	0.79–1.92	0.365	1.08	0.63–1.85	0.775
Corticosteroid use	1.33	0.90–1.95	0.152	1.28	0.79–2.08	0.313
Tonsillectomy	1.14	0.77–1.68	0.522	1.01	0.62–1.65	0.973

Univariate and multivariate analyses of factors related to greater percent change in eGFR (ΔeGFR% < -2.0 % per year) were performed on 416 patients, excluding one patient who subsequently became pregnant. The percent change in eGFR (ΔeGFR%) was calculated from the percentage of ΔeGFR in eGFR at the outpatient visit

immediately before vaccination. Categorical variables are presented as percentages and compared using Pearson's chi-square test. Clinically relevant factors identified by a subgroup comparison of patients and potential confounding factors, including a history of treatment for IgAN, such as corticosteroid treatment, renin-angiotensin-aldosterone system inhibitor treatment, and tonsillectomy, were included in the multivariate logistic regression analyses. Abbreviations: BMI, body mass index; CI, confidence interval; Cr, serum creatinine; eGFR, estimated glomerular filtration rate; IgAN, IgA nephropathy; OR, odds ratio; RAAS, renin-angiotensin-aldosterone system.
